# Identification of invasion-metastasis-associated microRNAs in hepatocellular carcinoma based on bioinformatic analysis and experimental validation

**DOI:** 10.1186/s12967-018-1639-8

**Published:** 2018-09-29

**Authors:** Weiyang Lou, Jing Chen, Bisha Ding, Danni Chen, Huilin Zheng, Donghai Jiang, Liang Xu, Chang Bao, Guoqiang Cao, Weimin Fan

**Affiliations:** 1 Program of Innovative Cancer Therapeutics, Division of Hepatobiliary and Pancreatic Surgery, Department of Surgery, First Affiliated Hospital, College of Medicine, Zhejiang University, 79 Qingchun Road, Hangzhou, 310003 China; 2 Key Laboratory of Organ Transplantation, Hangzhou, 310003 Zhejiang China; 3 Key Laboratory of Combined Multi-organ Transplantation, Ministry of Public Health, Hangzhou, 310000 China; 4 Department of Oncology, The First Hospital of Jiaxing, Jiaxing, 314000 Zhejiang China; 5 First Affiliated Hospital of Jiaxing University, Jiaxing, 314000 Zhejiang China; 6 Department of Pathology and Laboratory Medicine, Medical University of South Carolina, Charleston, SC 29425 USA

**Keywords:** Metastasis, Invasion, microRNA, Hepatocellular carcinoma, Bioinformatic analysis

## Abstract

**Background:**

Hepatocellular carcinoma (HCC) is one of the most lethal cancer, mainly attributing to its high tendency to metastasis. Vascular invasion provides a direct path for solid tumor metastasis. Mounting evidence has demonstrated that microRNAs (miRNAs) are related to human cancer onset and progression including invasion and metastasis.

**Methods:**

In search of invasion-metastasis-associated miRNAs in HCC, microarray dataset GSE67140 was downloaded from the Gene Expression Omnibus database. Differentially expressed miRNAs (DE-miRNAs) were obtained by R software package and the potential target genes were predicted by miRTarBase. The database for annotation, visualization and integrated discovery (DAVID) was introduced to perform functional annotation and pathway enrichment analysis for these potential targets of DE-miRNAs. Protein–protein interaction (PPI) network was established by STRING database and visualized by Cytoscape software. The effects of the miR-494-3p and miR-126-3p on migration and invasion of HCC cell lines were evaluated by conducting wound healing assay and transwell assay.

**Results:**

A total of 138 DE-miRNAs were screened out, including 57 upregulated miRNAs and 81 downregulated miRNAs in human HCC tumors with vascular invasion compared with human HCC tumors without vascular invasion. 762 target genes of the top three upregulated and downregulated miRNAs were predicted, and they were involved in HCC-related pathways, such as pathway in cancer, focal adhesion and MAPK signaling pathway. In the PPI network, the top 10 hub nodes with higher degrees were identified as hub genes, such as *TP53* and *MYC*. Through constructing the miRNA-hub gene network, we found that most of hub genes could be potentially modulated by miR-494-3p and miR-126-3p. Of note, miR-494-3p and miR-126-3p was markedly upregulated and downregulated in HCC cell lines and tissues, respectively. In addition, overexpression of miR-494-3p could significantly promote HCC migration and invasion whereas overexpression of miR-126-3p exerted an opposite effect.

**Conclusions:**

Targeting miR-494-3p and miR-126-3p may provide effective and promising approaches to suppress invasion and metastasis of HCC.

**Electronic supplementary material:**

The online version of this article (10.1186/s12967-018-1639-8) contains supplementary material, which is available to authorized users.

## Background

Hepatocellular carcinoma (HCC) is a high aggressive malignancy which mortality is nearly tantamount to its incidence [1]. It ranks the third most common causes of cancer-related death worldwide [2]. A variety of factors lead to the fearful situation, among which metastasis is the main one. Metastasis is a complicated process involving several sequential steps, including local invasion, intravasation, transport and survival in the circulatory system, extravasation, and settlement and proliferation in a new site [3]. As liver is an organ rich in blood vessel, vascular invasion is extremely frequent in clinical patients with HCC. Vascular invasion has a close connection with metastasis because it provides a direct path for cancer metastasis, and is a common precursor to occurrence of metastasis. Increasing studies have shown that cancer invasion and metastasis are closely associated with multiple biological processes, like degradation of extracellular matrix (ECM) [4], epithelial–mesenchymal transition (EMT) [5], immune evasion [6], homing of circulating cancer cells and cancer stem cells (CSCs) [7]. However, the detailed molecular mechanisms that manipulate invasion and metastasis of HCC are largely unknown and need to be further elucidated. Furthermore, most of effective targeted therapeutic drugs for HCC are unavailable.

MicroRNAs (miRNAs) are a group of small endogenous single-stranded non-coding RNAs, approximately 21–25 nucleotides in length [8]. MiRNAs can negatively modulate gene expression via binding to the 3′-untranslated region of messenger RNA (mRNA), thereby leading to direct degradation of mRNA or suppression of protein translation. Through this approach, miRNAs are involved in regulation of many biological processes such as proliferation, apoptosis, cell cycle and differentiation and DNA repair [9]. Over the past decades, mounting studies have demonstrated that miRNA is frequently abnormally expressed in various types of cancer including HCC [10], and the dysregulation of miRNA plays a paramount role in tumorigenesis, invasion and metastasis [11]. However, research exploring invasion-metastasis-associated miRNAs in HCC based on large-scale human tissues are rarely seen.

With the rapid development of gene chip and RNA sequencing technologies, Gene Expression Omnibus (GEO) gradually plays an important role in bioinformatic analysis [12]. It can provide us novel clues for discovering reliable and functional miRNAs. In this study, differentially expressed miRNAs (DE-miRNAs) in HCC samples with or without vascular invasion were screened out using miRNA expression profile of GSE67140. The potential target genes of the top three most upregulated and downregulated miRNAs were predicted by miRTarBase, and their potential functions were analyzed by gene ontology (GO) annotation and Kyoto Encyclopedia of Genes and Genomes (KEGG) pathway enrichment analysis. Moreover, a protein–protein interaction (PPI) network of these predicted target genes and miRNA-gene regulatory network were constructed by Cytoscape software. Next, the expression levels of the top 3 most upregulated and downregulated miRNAs in HCC cell lines were determined. Finally, we evaluated miR-494-3p and miR-126-3p on invasion and migration of HCC cell lines. The aim of present study is to explore and identify invasion-metastasis-associated miRNAs and their potential molecular mechanisms based on comprehensive bioinformatic analysis and related experimental validation.

## Methods

### MiRNA microarray

In the discovery step, we only included datasets that compared the miRNA expression in invasive/metastatic HCC cell or tissue with non-invasive/metastatic HCC cell or tissue. Besides, only datasets containing more than twenty samples were included. The titles and abstracts of these datasets were screened, and the full information of the datasets of interest were further evaluated. Finally, only GSE67140 dataset was selected to further study. The dataset GSE67140 based on the platform of GPL8786 (Affymetrix Multispecies miRNA-a Array) contained 91 human HCC tumor samples without vascular invasion and 81 samples with vascular invasion was downloaded from the National Center for Biotechnology Information (NCBI) GEO database (https://www.ncbi.nlm.nih.gov/geo).

### Screening for DE-miRNAs

**Fig. 1:**
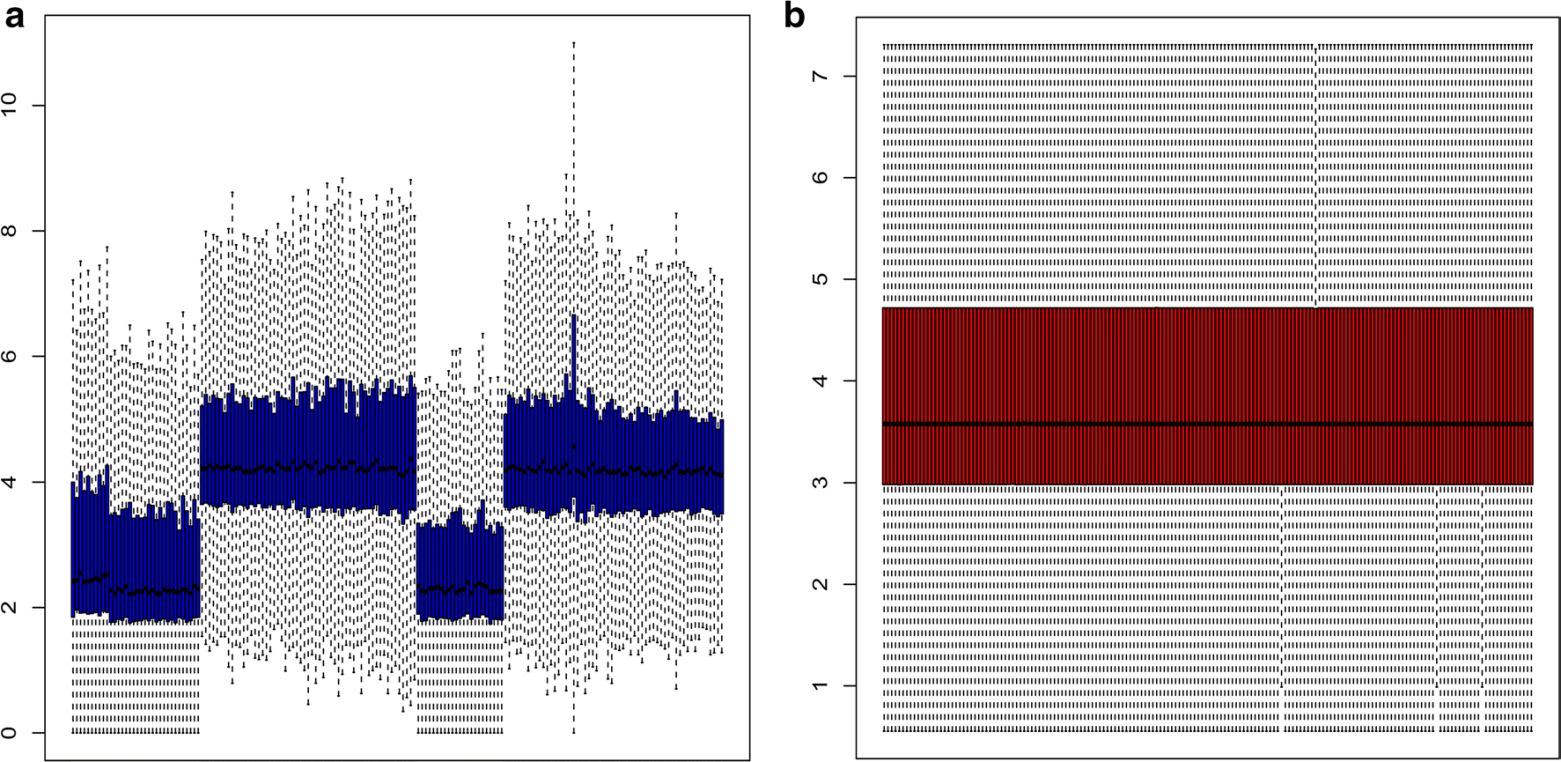
Normalization of GSE67140 data. **a** Data before normalization; **b** data after normalization

Data were normalized using the normalizeBetweenArray function from R package ‘LIMMA’ from the bioconductor project [13]. Data before and after normalization were shown in Fig. 1a, b, respectively. The miRNA differential expression analysis was conducted using the limma software package in the Bioconductor package (http://www.bioconductor.org/). The related codes were put into R, and the DE-miRNAs in HCC tumor samples with vascular invasion compared to HCC tumor samples without vascular invasion were analyzed through the limma package. *P*-value < 0.05 and |fold change (FC)| > 2 were set as the thresholds for identifying DE-miRNAs.

### Prediction of target genes

The potential target genes of the top three most upregulated and downregulated miRNAs were predicted by miRTarBase (http://mirtarbase.mbc.nctu.edu.tw/php/index.php), which is an experimentally validated microRNA-target interactions database [14].

### GO and pathway analysis

The database for annotation, visualization and integrated discovery (DAVID 6.8, http://david-d.ncifcrf.gov/) was introduced to perform functional annotation and pathway enrichment analysis for the predicted targets of the selected 6 DE-miRNAs, including GO and KEGG pathway analysis [15, 16]. *P*-value < 0.05 was considered as statistically significant.

### PPI network and miRNA-gene network construction

PPI network and miRNA-gene network were successively constructed. The target genes were first mapped to the STRING database (http://string-db.org) to assess functional associations among these target genes [17]. Only the interactions with a combined score > 0.4 were considered as significant. Next, to obtained the hub genes, the degree of connectivity in the PPI network was analyzed by Cytoscape software (version 3.6.0), after which miRNA-hub gene network was established.

### Cell lines and clinical samples

The human HCC cell lines HepG2, Bel-7402, HCCLM3 and SMMC7721 and normal cell line HL7702 were kindly provided by the First Affiliated Hospital of Medical College, Zhejiang University (Hangzhou, China). HepG2, Bel-7402, HCCLM3 and HL7702 were cultured in Dulbecco’s modified Eagle’s medium (DMEM; Gibco,12430047) supplemented with 10% fetal bovine serum (FBS; Biological Industries, 04-0101-1, Cromwell, CT, USA) and SMMC7721 was maintained in Roswell Park Memorial Institute (RPMI) 1640 medium (Gibco, 31800105, Life Technologies, Carlsbad) containing 10% FBS under a humidified atmosphere of 5% CO_2_ at 37 °C. HCC tumor tissues and matched noncancerous tissues were also obtained from the First Affiliated Hospital of Medical College, Zhejiang University (Hangzhou, China).

### Cell transfection

The miRNA mimics and negative control (purchased from RiboBio, Guangzhou, China) were transfected into HCC cell lines using Lipofectamine™ 3000 according to the manufacturer’s instruction.

### RNA extraction and quantitative-PCR (qPCR)

RNAiso plus Reagent (TaKaRa, Kusatsu, Japan) was introduced to extract total RNA from HCC cell lines and clinical samples. Subsequently, miRNAs reverse transcription primers and q-PCR primers were purchased from Ribobio Co. Ltd (Guangzhou, China). Q-PCR was performed in triplicates by SYBR Premix Ex Taq (TaKaRa, RR420A). The U6 small nuclear RNA was used as the internal control. The 2^−ΔΔCT^ method was utilized to determine fold change in the RNA level of each sample compared with the reference sample.

### The Cancer Genome Atlas database

The expression levels of miR-494-3p and miR-126-3p in HCC tissue from the Cancer Genome Atlas (TCGA) database (https://genome-cancer.ucsc.edu/) were determined. Firstly, the miRNA-sequencing data in patients of liver cancer was downloaded from the database. Then, miR-494-3p and miR-126-3p expression values were extracted, after which unpaired Student’s *t*-test was employed to conduct statistical analysis between normal group and tumor group. A *P*-value < 0.05 was considered as significant.

### Wound healing assays

Cells were plated in six-well plates with 1 × 10^5^ cells per well. After transfection (50 nM), the cells were grown to 100% confluence in six-well plates. Then, a micropipette tip was applied to make a cross wound. Photographs were taken by a microscopy immediately or 24 h after wounding.

### Transwell assay

The cell invasion assay was performed using 24-well transwell chambers (Corning, USA). Firstly, the inserts were coated with Matrigel (BD Bioscience, USA) on the upper surface. After transfection (50 nM), 1 × 10^5^ cells were suspended in 0.2 ml serum-free medium and added to the inserts. 0.6 ml medium with 20% FBS was added to the lower compartment as a chemoattractant. After incubation at 37 °C for 48 h, the cells on the upper surface of the membrane were carefully removed using a cotton bud and cells on the lower surface were successively fixed with 100% methanol and stained with 0.1% crystal violet. Five visual fields of 200× magnification of each insert were randomly selected and counted under a light microscope (Olympus, Japan).

### Analysis of target gene expression using UALCAN database

UALCAN, a portal for facilitating tumor subgroup gene expression and survival analyses, provides easy access to publicly available cancer transcriptome data including TCGA [18]. The database was utilized to analyze the expression levels of potential targets of miR-494-3p and miR-126-3p between normal tissue and tumor tissue. In brief, gene symbols were firstly pasted in the text area, and liver hepatocellular carcinoma dataset was chosen. Then, the expression information of each target gene could be acquired by linking to gene expression analysis result. Statistical analysis of comparison between normal group and tumor group was performed and log-rank *P*-value was observed in the database.

### Survival data from Kaplan–Meier plotter

The prognostic values of miR-494-3p and miR-126-3p in HCC were analyzed using Kaplan–Meier plotter (KM plotter) database [19]. In brief, the two miRNAs were entered into the database, after which Kaplan–Meier survival plots were generated and hazard ratio (HR), 95% confidence intervals (CI), log rank *P*-value were displayed on the webpage. *P*-value of < 0.05 was regarded as statistically significant.

### Statistical analysis

The results were shown as mean ± SD. Differences between two groups were estimated using unpaired Student’s *t*-test. A two-tailed value of *P *< 0.05 was considered as statistically significant.

## Results

### Identification of DE-miRNAs and their target genes

**Fig. 2:**
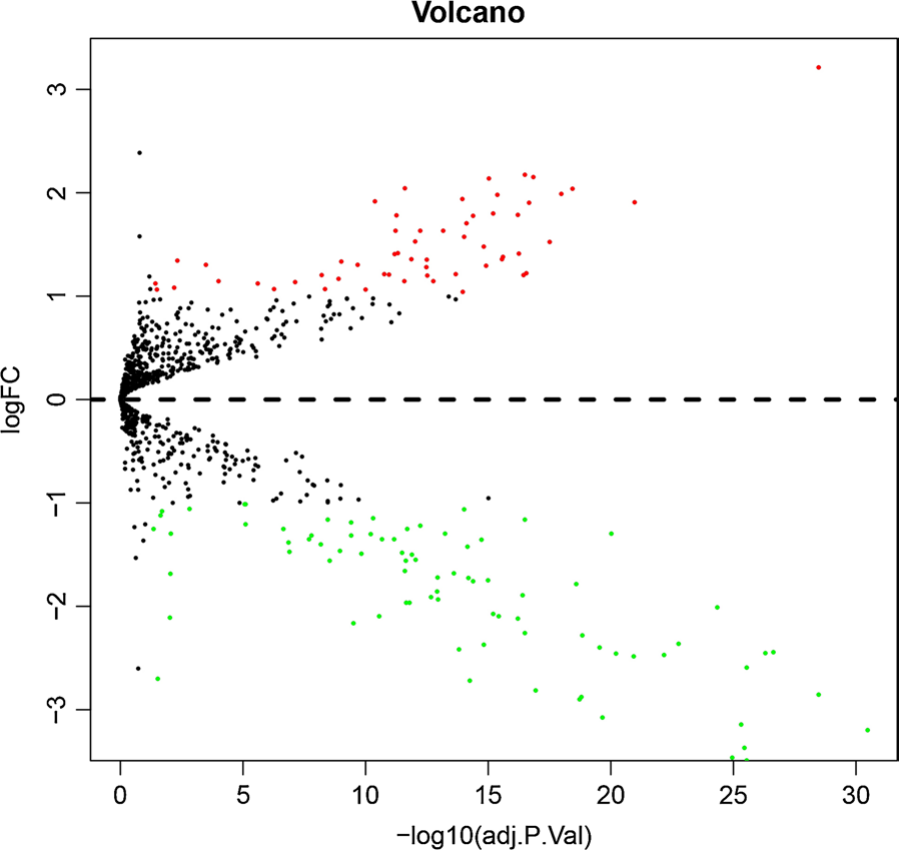
Volcano plot of the DE-miRNAs. The black dots represent miRNAs that are not differentially expressed between 91 human HCC tumor samples without vascular invasion and 81 samples with vascular invasion, and the red dots and green dots represent the upregulated and downregulated miRNAs in HCC tumor samples with vascular invasion, respectively

To identify DE-miRNAs of miRNA array GSE67140 downloaded from GEO, we conducted a differential expression analysis using limma software package. Based on this analysis, a total of 138 miRNAs were found to be significantly differentially expressed in HCC tumor samples with vascular invasion more than two-fold when compared to HCC tumor samples without vascular invasion, including 57 upregulated and 81 downregulated miRNAs. For better visualization, the top ten most upregulated miRNAs and top ten most downregulated miRNAs were presented in Tables 1 and 2, respectively. According to fold change (FC), miR-494-3p, miR-1207-5p and miR-1268a were the top three most upregulated miRNAs; and miR-126-3p, miR-199a-3p and miR-199b-3p were the top three most downregulated miRNAs. 485 potential target genes were predicted for the three upregulated miRNAs and 277 genes for the three downregulated miRNAs by using miRTarBase. A volcano plot of these DE-miRNAs was provided in Fig. 2.

**Table 1:** Top ten upregulated differentially expressed miRNAs between HCC tumor with vascular invasion and without vascular invasion

miRNA	Log_2_FC	AveExpr	t	P value	adj.P.val	B
hsa-miR-494-3p	3.213965	8.547004	14.52827	9.49E−32	3.34E−29	61.55024
hsa-miR-1207-5p	2.176223	9.115458	9.917775	1.25E−18	3.21E−17	31.61977
hsa-miR-1268a	2.152237	8.776311	10.0675	4.80E−19	1.45E−17	32.56622
hsa-miR-1225-5p	2.137299	7.255844	9.334773	5.00E−17	9.42E−16	27.97336
hsa-miR-923	2.043838	11.34486	7.931558	2.61E−13	2.51E−12	19.52928
hsa-miR-1281	2.038617	7.431229	10.66035	1.05E−20	3.72E−19	36.34667
hsa-miR-1275	1.991306	6.77929	10.49143	3.14E−20	1.07E−18	35.26462
hsa-miR-1224-5p	1.982613	5.940673	9.473018	2.10E−17	4.24E−16	28.83197
hsa-miR-92b-5p	1.93886	7.353231	8.887235	8.10E−16	1.13E−14	25.22309
hsa-miR-572	1.917433	6.686904	7.419681	5.11E−12	4.21E−11	16.60168

**Table 2:** Top ten downregulated differentially expressed miRNAs between HCC tumor with vascular invasion and without vascular invasion

miRNA	Log_2_FC	AveExpr	t	P value	adj.P.val	B
hsa-miR-199a-3p	− 3.49273	7.052915	− 13.3399	2.38E−28	2.88E−26	53.79083
hsa-miR-199b-3p	− 3.46525	7.057111	− 13.0789	1.33E−27	1.13E−25	52.08226
hsa-miR-126-3p	− 3.36828	9.212248	− 13.2869	3.38E−28	3.58E−26	53.44345
hsa-miR-195-5p	− 3.19533	7.128742	− 15.3637	4.02E−34	3.41E−31	66.96628
hsa-miR-30a-5p	− 3.14494	7.071084	− 13.2253	5.07E−28	4.78E−26	53.04028
hsa-miR-143-3p	− 3.07332	9.16678	− 11.141	4.62E−22	2.17E−20	39.44342
hsa-miR-100-5p	− 2.90109	8.128079	− 10.7776	4.92E−21	1.90E−19	37.10007
hsa-miR-19b-3p	− 2.87469	7.51099	− 10.8113	3.96E−21	1.60E−19	37.31624
hsa-miR-21-5p	− 2.85469	5.834845	− 14.4948	1.18E−31	3.34E−29	61.33273
hsa-miR-146b-5p	− 2.81179	6.432729	− 10.1073	3.72E−19	1.17E−17	32.81846

### GO functional enrichment analysis

**Fig. 3:**
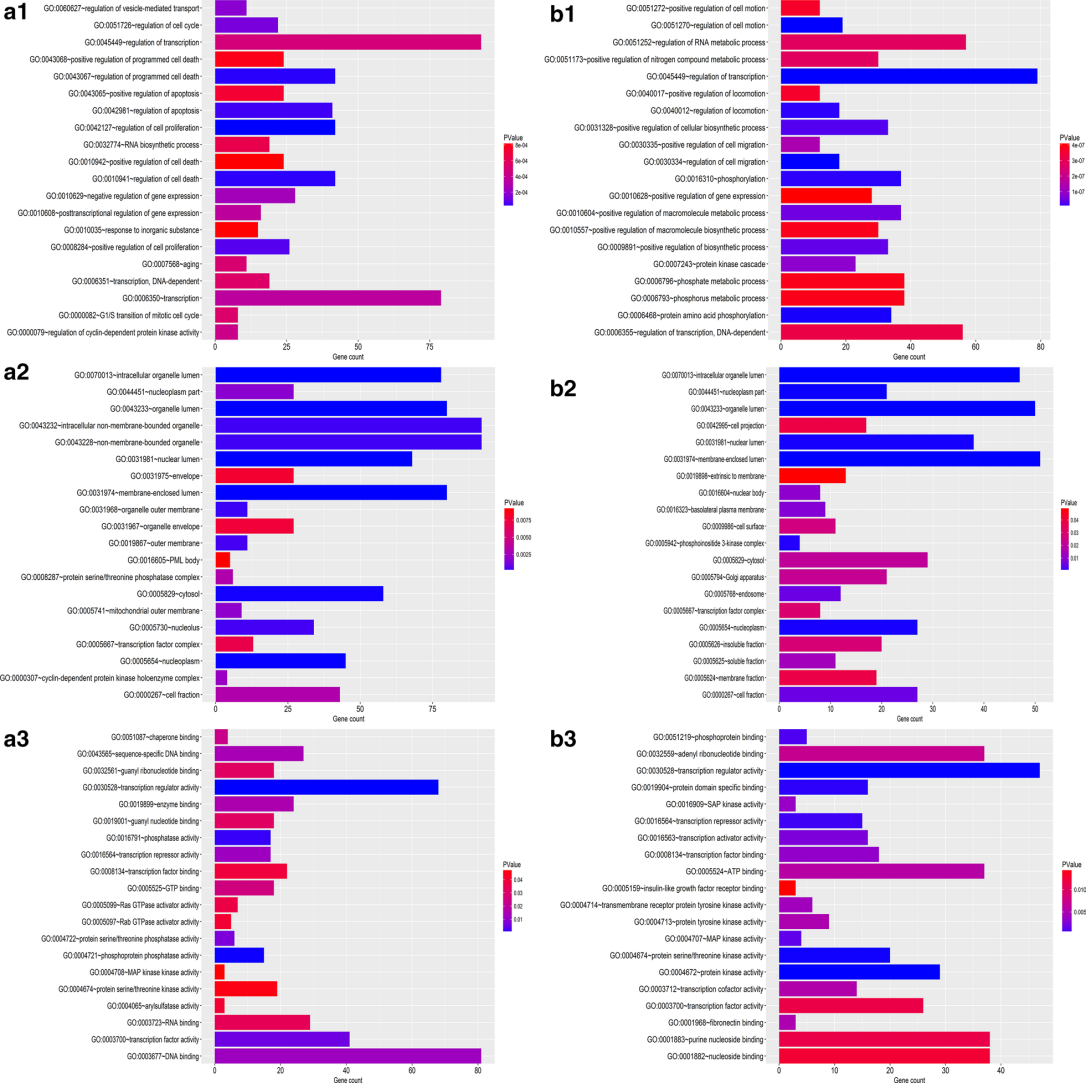
GO functions for the target genes of top three upregulated miRNAs and top three downregulated miRNAs. **a1** Enriched biological process of the upregulated miRNAs; **a2** enriched cellular component of the upregulated miRNAs; **a3** enriched molecular function of the upregulated miRNAs; **b1** enriched biological process of the downregulated miRNAs; **b2** enriched cellular component of the downregulated miRNAs; **b3** enriched molecular function of the downregulated miRNAs

Three categories of GO functional annotation analysis were performed on these potential target genes mentioned above, including biological process (BP), cellular component (CC) and molecular function (MF). As shown in Fig. 3a1–a3, the enriched GO functions for target genes of the three upregulated miRNAs included the regulation of transcription, transcription, regulation of programmed cell death, regulation of cell death and regulation of apoptosis in the BP category; intracellular non-membrane-bounded organelle, non-membrane-bounded organelle, membrane-enclosed lumen, organelle lumen and intracellular organelle lumen in the CC category; and DNA binding, transcription regulator activity, transcription factor binding, RNA binding and sequence-specific DNA binding in the MF category. The enriched GO functions for target genes of the three downregulated miRNAs were presented in Fig. 3b1–b3, involving the regulation of transcription, regulation of RNA metabolic process, positive regulation of cellular biosynthetic process, positive regulation of macromolecule metabolic process and phosphorylation in the BP category; membrane-enclosed lumen, organelle lumen, intracellular organelle lumen, nuclear lumen and cytosol in the CC category; and transcription regulator activity, adenyl ribonucleotide binding, ATP binding, purine nucleoside binding and nucleoside binding in the MF category.

### KEGG pathway enrichment analysis

**Fig. 4:**
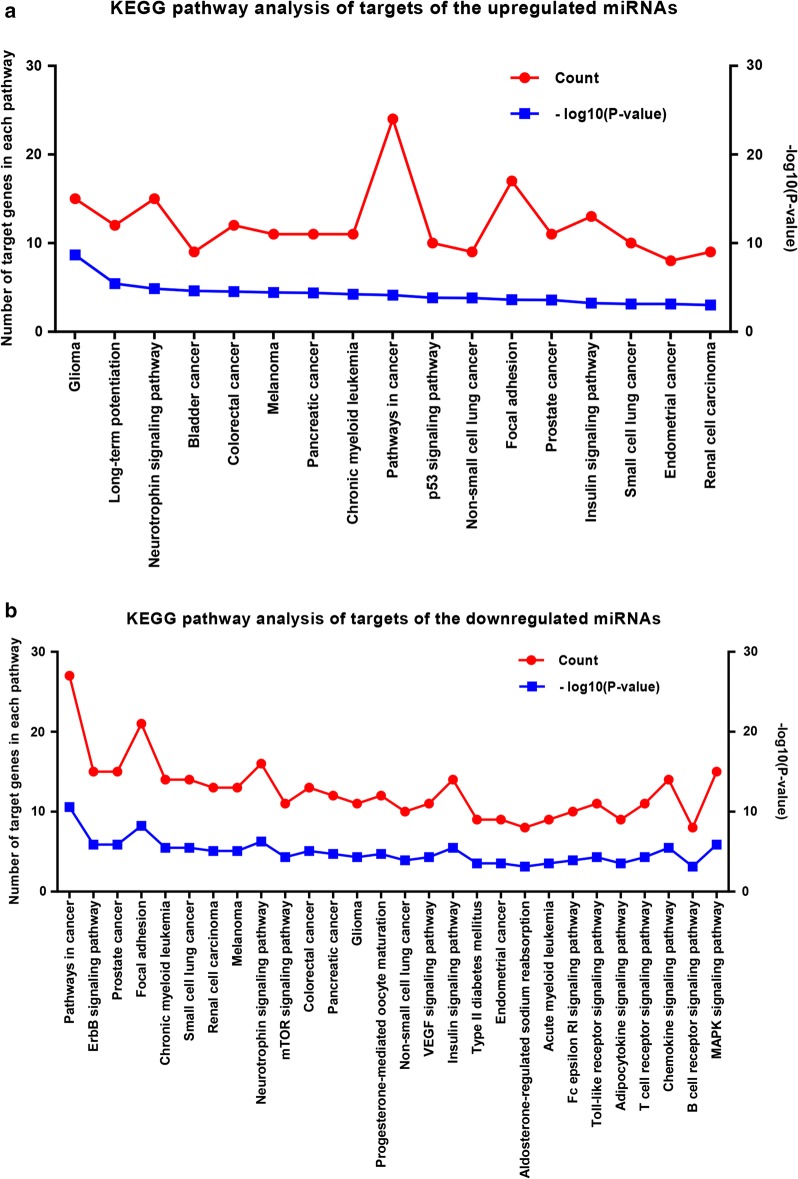
KEGG pathway enrichment analysis of target genes of six selected DE-miRNAs. **a** For upregulated miRNAs; **b** for downregulated miRNAs. Only the enriched pathways with *P*-value < 0.001 were presented. The red lines represent count and the blue lines represent − log_10_(P-value)

To further analyze the enriched pathways of these target genes, we subsequently conducted KEGG pathway enrichment analysis. For upregulated miRNAs (Fig. 4a), the enriched KEGG pathways contained pathways in cancer, focal adhesion, glioma, neurotrophin signaling pathway and insulin signaling pathway. And the enriched KEGG pathways for downregulated miRNAs (Fig. 4b) included pathways in cancer, focal adhesion, MAPK signaling pathway, neurotrophin signaling pathway and chemokine signaling pathway. Interestingly, both target genes of upregulated miRNAs and targets of downregulated miRNAs were enriched in the process, focal adhesion. Accumulating evidence has demonstrated that dysregulation of focal adhesion plays a central role in cell invasion and migration [20]. Therefore, 17 genes (*PRKCA*, *HRAS*, *TLN1*, *MAP2K1*, *PPP1CC*, *PTEN*, *AKT1*, *VEGFB*, *MAPK1*, *IGF1R*, *CCND1*, *ARHGAP5*, *BCL2*, *RAC1*, *PPP1R12A*, *RAP1A*, *RAP1B*) of upregulated miRNAs and 21 genes (*PIK3CG*, *CAV2*, *FLT1*, *ROCK1*, *MET*, *IGF1*, *ITGA3*, *HGF*, *KDR*, *AKT1*, *MAPK1*, *CRKL*, *PAK4*, *BCL2*, *VEGFA*, *MAPK9*, *MAPK8*, *CRK*, *PIK3R1*, *AKT2*, *PIK3R2*) of downregulated miRNAs which are involved in focal adhesion may interact with the selected 6 miRNAs, thereby modulating invasion and metastasis in HCC.

### Construction and analysis of PPI network and miRNA-hub gene network

The PPI networks of target genes of the six selected DE-miRNAs were constructed using the STRING database. Data from this database revealed that lots of these target genes could interact with each other. The top ten hub genes listed in Table 3 were screened out according to the node degree. For the upregulated miRNAs, the hub genes were *TP53*, *AKT1*, *MAPK1*, *MYC*, *BCL2*, *HRAS*, *PTEN*, *CALM3*, *CDKN1A*, *CYCS*. For the downregulated miRNAs, the hub genes were *MYC*, *AKT1*, *VEGFA*, *MAPK8*, *PIK3CG*, *BCL2*, *FGF2*, *KRAS*, *PIK3R1*, *SIRT1*. Among these genes, *TP53* and *MYC* demonstrated the highest node degrees, which were 80 and 42, respectively. The results suggest that *TP53* and *MYC* may be two key targets correlated with HCC invasion and metastasis.

**Table 3:** Hub genes identified in the PPI interaction

Upregulated miRNAs		Downregulated miRNAs	
Gene symbol	Degree	Gene symbol	Degree
TP53	80	MYC	42
AKT1	72	AKT1	39
MAPK1	61	VEGFA	38
MYC	51	MAPK8	35
BCL2	46	PIK3CG	35
HRAS	46	BCL2	28
PTEN	46	FGF2	22
CALM3	45	KRAS	22
CDKN1A	44	PIK3R1	21
CYCS	40	SIRT1	21

*PPI* protein–protein interaction

**Fig. 5:**
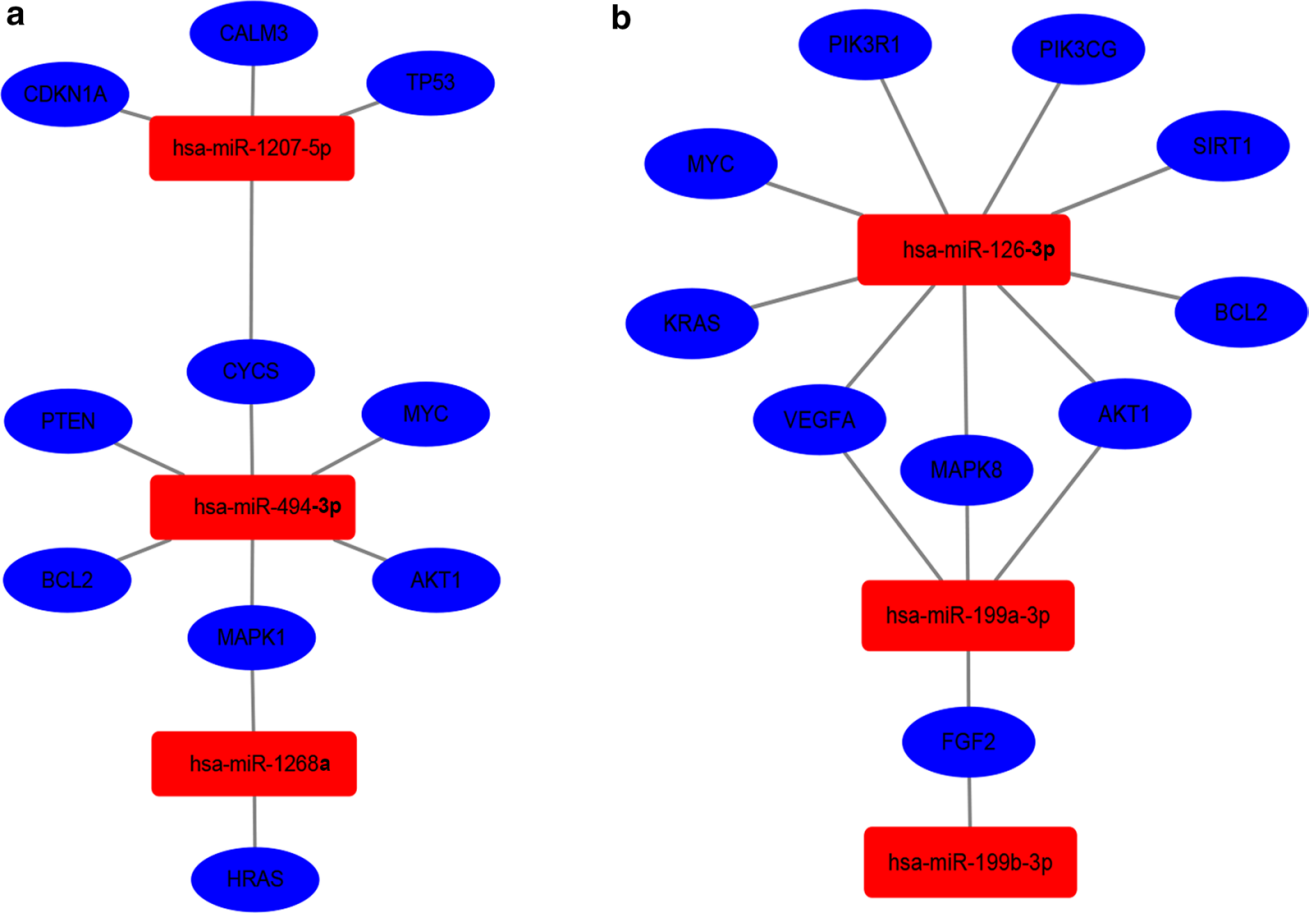
The regulatory network between dysregulated miRNAs and hub genes. **a** For upregulated miRNAs; **b** for downregulated miRNAs

Subsequently, the miRNA-hub gene network was constructed by cytoscape software as showed in Fig. 5. From Fig. 5a, we found that six (*CYCS*, *MYC*, *AKT1*, *MAPK1*, *BCL2* and *PTEN*) in ten hub genes could be potentially modulated by upregulated miR-494-3p. 4 hub genes and 2 hub genes could be potentially regulated by miR-1207-5p and miR-1268a, respectively. Additionally, miR-126-3p could potentially target nine (*PIK3R1*, *PIK3CG*, *SIRT1*, *BCL2*, *AKT1*, *MAPK8*, *VEGFA*, *KRAS* and *MYC*) in ten hub genes. And 4 hub genes and 1 hub gene could be potentially modulated by miR-199a-3p and miR-199b-3p. These data supported that miR-494-3p and miR-126-3p might be two potential regulators of invasion and metastasis in HCC.

**Fig. 6:**
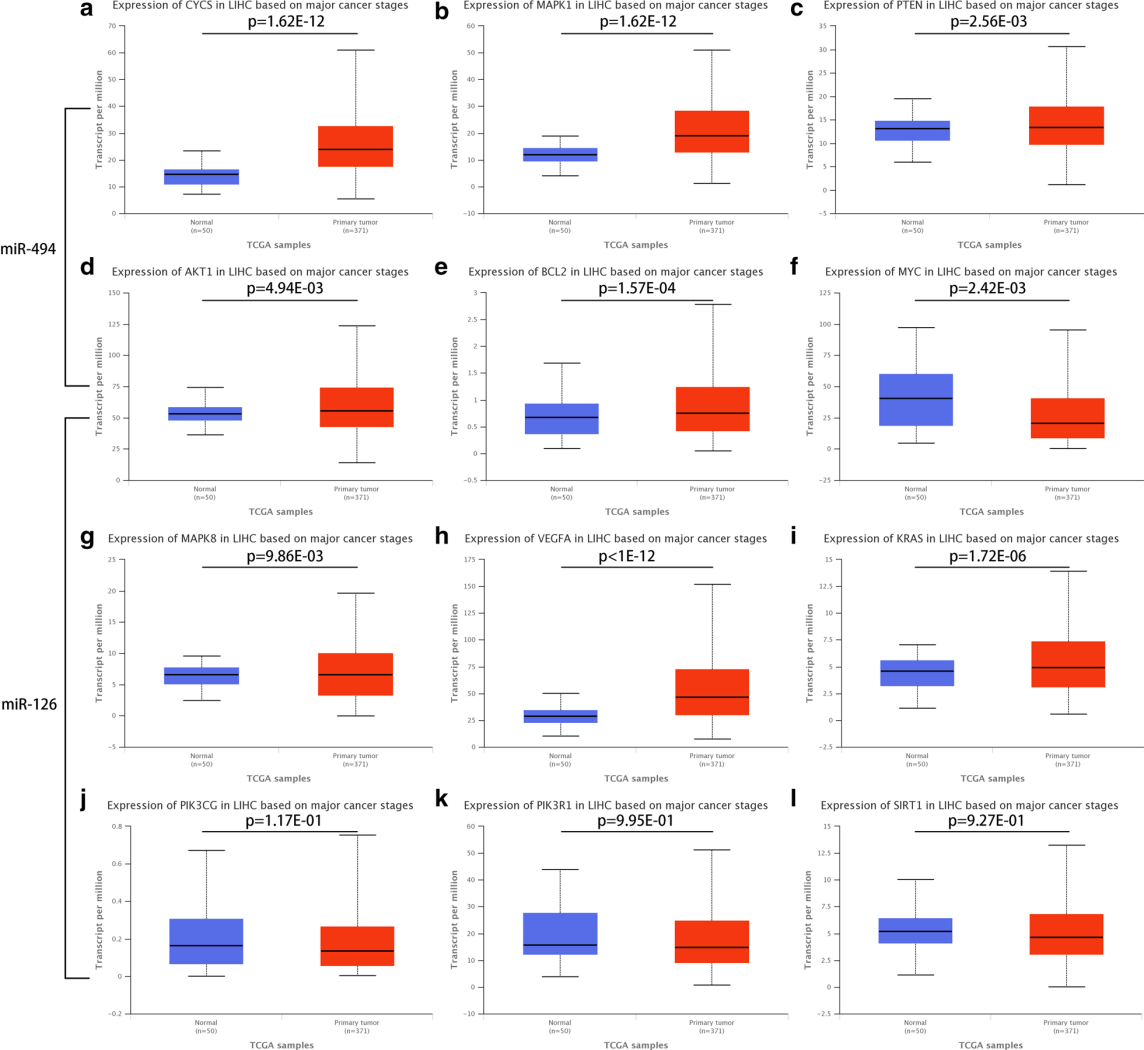
The mRNA expression of predicted targets of miR-494-3p and miR-126-3p from the UALCAN database. **a** *CYCS* expression; **b** *MAPK1* expression; **c** *PTEN* expression; **d** *AKT1* expression; **e** *BCL2* expression; **f** *MYC* expression; **g** *MAPK8* expression; **h** *VEGFA* expression; **i** *KRAS* expression; **j** *PIK3CG* expression; **k** *PIK3R1* expression; **l** *SIRT1* expression

Next, we further evaluated the expression of potential targets of miR-494-3p and miR-126-3p in HCC using the UALCAN database as shown in Fig. 6. The analytic results suggested that the expression of five (*CYCS*, *MAPK1*, *PTEN*, *AKT1*, *BCL2*) in six targets of miR-494 was significantly upregulated in HCC tissue than normal tissue whereas *MYC* gene was markedly downregulated. It is widely acknowledged that there is an inverse relationship between miRNA expression and target gene expression. Thus, among these detected genes, the significantly downregulated *MYC* may be the most potential target for the upregulated miR-494-3p. Similarly, these significantly upregulated genes including *AKT1*, *BCL2*, *MAPK8*, *VEGFA* and *KRAS* may be the most potential targets for the downregulated miR-126-3p in theory.

### Aberrant expression and prognostic roles of miR-494-3p and miR-126-3p in HCC

**Fig. 7:**
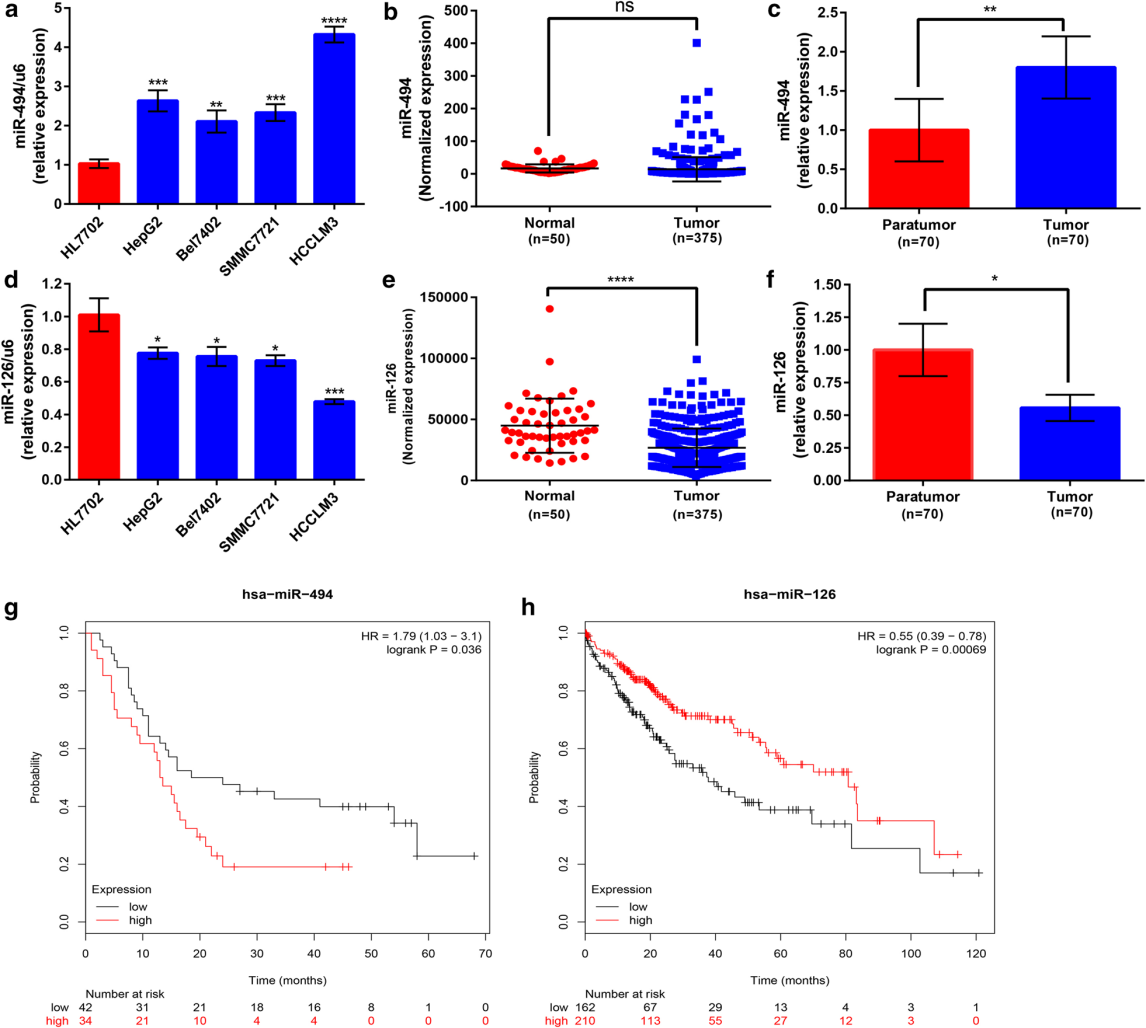
The expression and prognostic roles of miR-494-3p and miR-126-3p in HCC. **a** The expression of miR-494-3p in four HCC cell lines (HepG2, Bel7402, SMMC7721, HCCLM3) was compared with that in one liver cell line (HL7702); **b** the expression of miR-494-3p in tumor tissues was compared with that in normal tissues in HCC patients from TCGA database; **c** the miR-494-3p expression in tumor tissues was compared with adjacent normal tissues in HCC patients from clinical; **d** the expression of miR-126-3p in four HCC cell lines (HepG2, Bel7402, SMMC7721, HCCLM3) was compared with that in HL7702; **e** the expression of miR-126-3p in tumor tissues was compared with that in normal tissues in HCC patients from TCGA database; **f** the miR-126-3p expression in tumor tissues was compared with adjacent normal tissues in HCC patients from clinical; **g** Kaplan–Meier survival curve of miR-494-3p in HCC; **h** Kaplan–Meier survival curve of miR-126-3p in HCC

Based on our previous findings, we intended to further determine the expression and prognostic roles of miR-494-3p and miR-126-3p in HCC. Firstly, we evaluated the expression of miR-494-3p and miR-126-3p in four HCC cell lines (HepG2, Bel7402, SMMC7721 and HCCLM3) compared with that in normal liver cell line (HL7702). The results presented in Fig. 7a, d demonstrated that miR-494-3p expression was markedly upregulated and miR-126-3p expression were significantly downregulated in all four HCC cell lines. Of note, expression of miR-494-3p and miR-126-3p in HCCLM3 is the most upregulated and downregulated compared with other three cell lines. HCCLM3 is a high metastatic HCC cell [21], further suggesting miR-494-3p and miR-126-3p may regulate HCC metastasis. The expression of miR-1207-5p, miR-1268a, miR-199a-3p and miR-199b-3p in HCC cell lines was also detected (Additional file 1 : Figure S1). However, the expression of them was only significantly dysregulated in one or two HCC cell lines as shown in Additional file 1 : Figure S1. To some extent, the result hinted that the functional roles of miR-1207-5p, miR-1268a, miR-199a-3p and miR-199b-3p in HCC may be not as good as miR-494-3p and miR-126-3p. Besides, miR-494-3p and miR-126-3p expression in 70 pairs of clinical tumor and normal samples also demonstrated a higher expression of miR-494-3p and a lower expression of miR-126-3p in HCC tissues than normal tissues (Fig. 7c, f), further hinting promoting and suppressive roles of miR-494-3p and miR-126-3p in HCC, respectively. Moreover, for miR-126-3p, similar results were obtained by downloading and analyzing HCC data from the Cancer Genome Atlas (TCGA) (Fig. 7e). However, for miR-494-3p, there was no significant difference between tumor tissue and normal tissue (Fig. 7b). To summarize, miR-494-3p was upregulated and miR-126-3p was downregulated in HCC, suggesting that they may play a major role in the onset and progression of HCC, especially in HCC invasion and metastasis.

KM plotter database was utilized to assess the prognostic values of the two potential miRNAs, miR-494-3p and miR-126-3p in HCC. As shown in Fig. 7g, high expression of miR-494-3p was significantly correlated with worse OS, further implying that miR-494-3p is an oncogene in HCC. Figure 7h indicated a favorable prognostic role of miR-126-3p in HCC. In short, all these findings suggest that miR-494-3p and miR-126-3p are aberrantly expressed in HCC and can serve as two prognostic biomarkers. Therefore, the two miRNAs, miR-494-3p and miR-126-3p were selected for further experimental excavation and research.

### In vitro effects of miR-494-3p and miR-126-3p on HCC migration and invasion

**Fig. 8:**
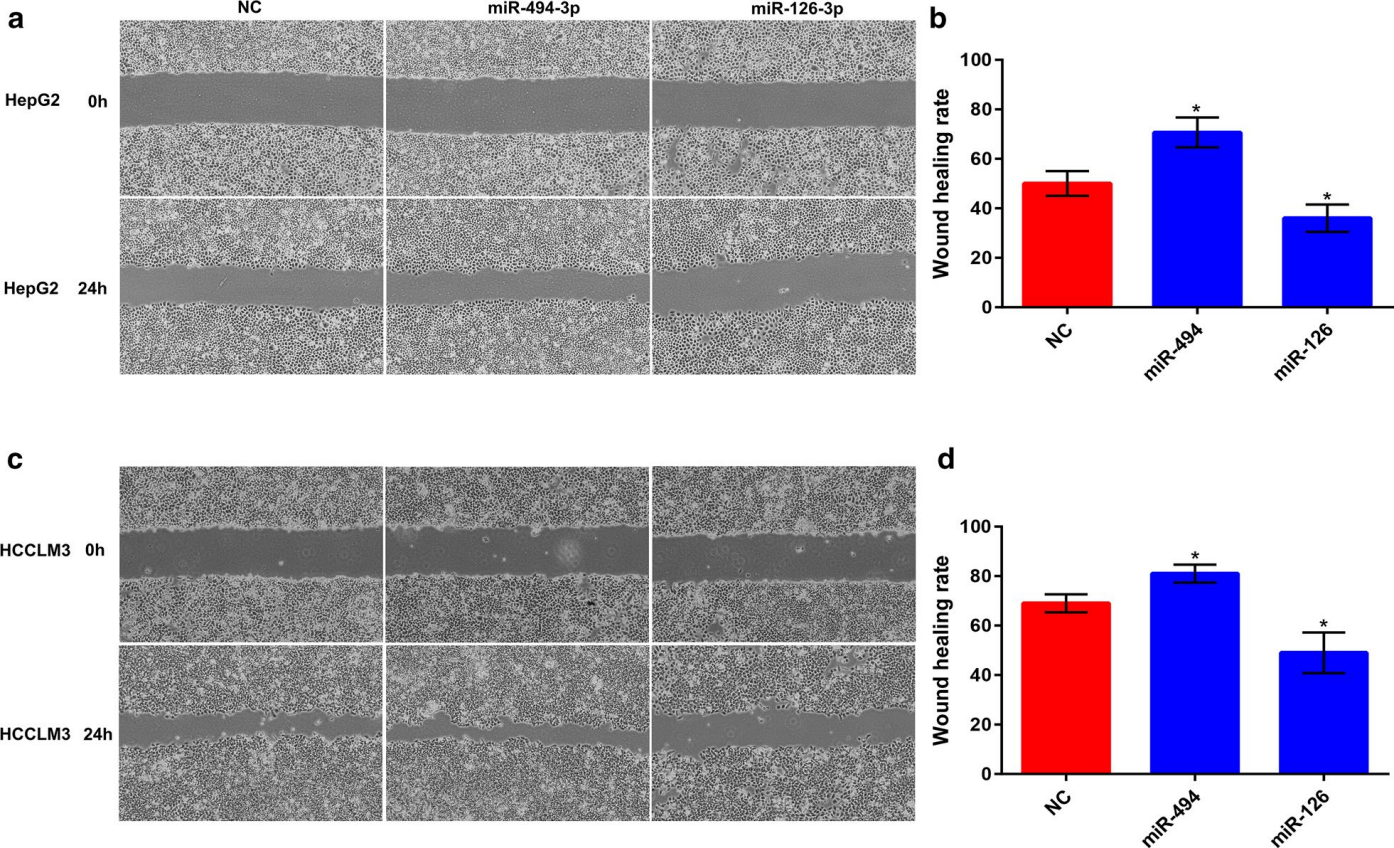
Overexpression of miR-494-3p and miR-126-3p regulate HCC cell motility. **a** HepG2 was transfected with NC, miR-494-3p mimic, miR-126-3p mimic, respectively. Wound healing assay was performed with a 24-h recovery period; **b** quantification of motility from **a**; **c** HCCLM3 was transfected with NC, miR-494-3p mimic, miR-126-3p mimic, respectively. Wound healing assay was performed with a 24-h recovery period; **d** quantification of motility from **a**

**Fig. 9:**
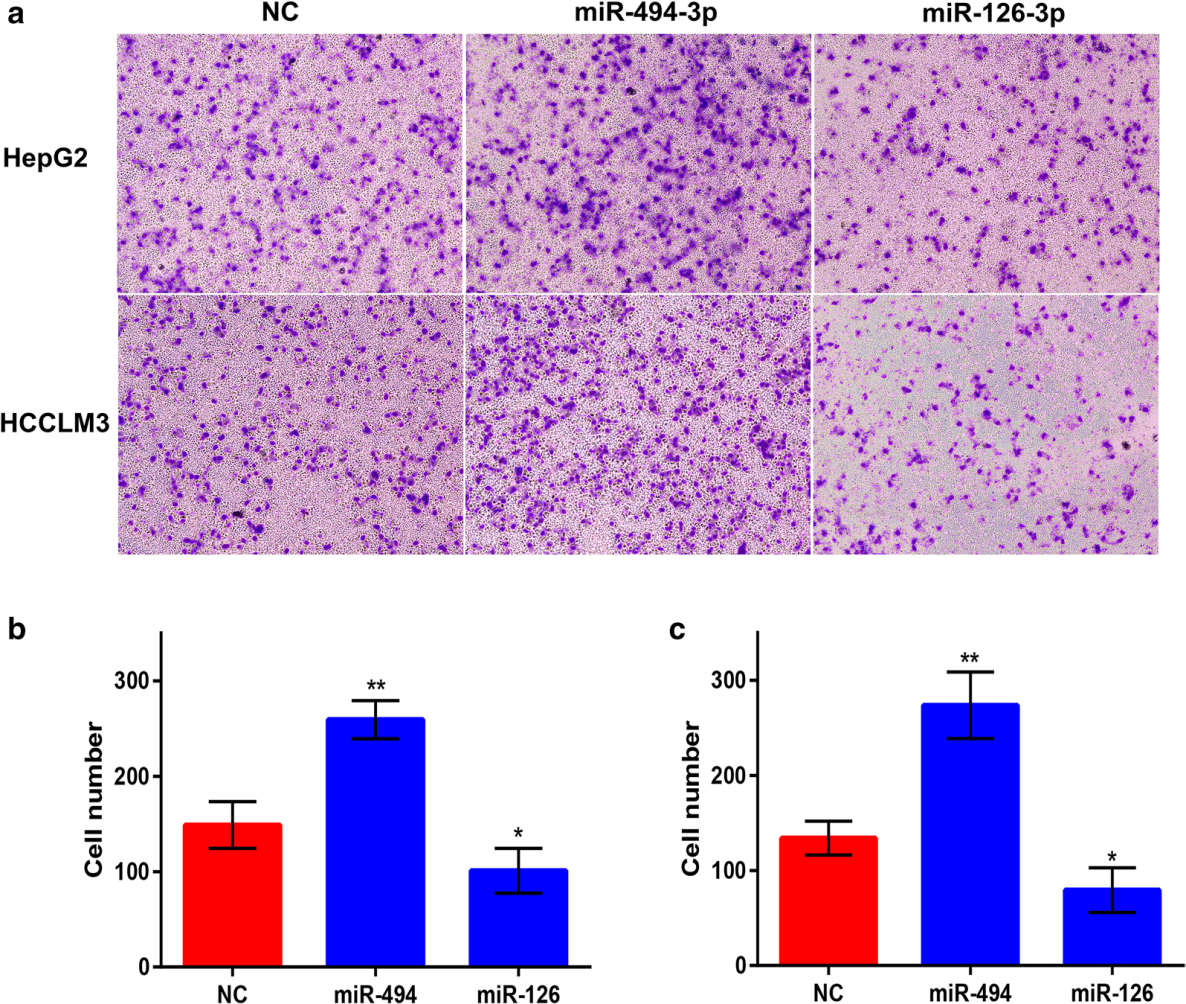
Overexpression of miR-494-3p and miR-126-3p regulate HCC cell invasion. **a** HepG2 and HCCLM3 transfected with miR-494-3p invaded more versus control cancer cells, whereas HepG2 and HCCLM3 transfected with miR-126-3p invaded less versus control cancer cells; **b** quantification of invasion from HepG2; **c** quantification of invasion from HCCLM3

In the first place, miR-494-3p and miR-126-3p expression in HepG2 and HCCLM3 after transfected with miRNA mimics at final concentrations of 20, 50 and 100 nM were detected using qPCR (Additional file 2 : Figure S2). To determine the roles of miR-494-3p and miR-126-3p in HCC migration, we first performed a wound healing assay. As shown in Fig. 8, wound healing assay suggested that miR-494-3p overexpression markedly enhanced HepG2 and HCCLM3 migration whereas overexpression of miR-126-3p could significantly attenuate cell migration, when compared with negative control cells. Next, a transwell invasion assay was employed to measure the effects of miR-494-3p and miR-126-3p on HCC invasion. The results presented in Fig. 9 demonstrated that upregulation of miR-494-3p and miR-126-3p could evidently boost and suppress cell invasion, respectively. All these data confirmed that miR-494-3p and miR-126-3p were two key regulators of HCC invasion and metastasis. Targeting miR-494-3p and miR-126-3p may represent novel approaches in treating HCC patients with vascular invasion or/and distant metastasis, thus improving the prognosis of patients with HCC.

## Discussion

HCC, a high heterogeneous malignancy, which prognosis is extremely dismal, mainly attributing to its tendency to invade and metastasize. To date, effective therapeutic strategies against invasive or/and metastatic HCC is still rare. Therefore, it is imperative need to seek and develop a promising therapeutic target to improve the survival of patients with advanced HCC.

MiRNAs are a group of small, endogenous non-coding RNAs which possess a variety of biological functions, including modulating cell metabolism and cell survival [22, 23]. Recent studies showed that miRNA is involved in HCC invasion and metastasis by post-transcriptionally regulating gene expression. For example, the team of Liao CG found that miR-10b promotes migration and invasion of HCC via direct repression of RhoC, uPAR and MMPs [24]. In addition, Yang et al. [25] suggested that miR-34a is involved in Treg cell recruitment and promotes venous metastases of HBV-positive hepatocellular carcinoma. However, systemic analysis of miRNA’s roles in HCC invasion and metastasis remain absent.

In this study, we discovered some invasion-metastasis-associated miRNAs by performing a differential expression analysis on a microRNA array downloaded from GEO datasets. According to node degree, top ten hub genes of three upregulated miRNAs and three downregulated miRNAs were screened out. By constructing miRNA-gene network, we found that most of hub genes could be potentially modulated by miR-494-3p and miR-126-3p. Recent studies have suggested that miR-494-3p is linked to the occurrence and development of various types of cancer, including nasopharyngeal carcinoma, oral cancer, non-small cell lung cancer, gastric cancer and osteosarcoma [26–30]. MiR-126-3p is also reported to suppress cancer proliferation, migration and increase chemosensitivity [31–34]. To the best of our knowledge, however, the roles of miR-494-3p and miR-126-3p in HCC, especially in invasion and metastasis of HCC, have not been studied. It is extremely meaningful to authenticate the functions of miR-494-3p and miR-126-3p in HCC and elucidate the mechanisms how they regulate HCC invasion and metastasis.

Q-PCR data revealed that miR-494-3p and miR-126-3p were significantly upregulated and downregulated in both cell lines and clinical samples. Subsequently, in vitro wound healing assay and transwell invasion assay suggested that overexpression of miR-494-3p and miR-126-3p could markedly promote and suppress HCC migration and invasion, respectively. In addition, GO annotation and KEGG pathway analysis on the predicted target genes of the top three most upregulated and downregulated miRNAs were conducted using DAVID, and the results demonstrated that some of these target genes were enriched in focal adhesion. It has been well documented that focal adhesion is closely associated with cell migration and invasion [20]. Therefore, targeting these genes may be one of the mechanisms that miR-494-3p and miR-126-3p exert their effects on invasion and metastasis in HCC.

In a word, we confirmed that miR-494-3p and miR-126-3p are aberrantly expressed in both HCC cell lines and clinical samples and are two crucial regulators of HCC invasion and metastasis and can serve as prognostic biomarkers in HCC. However, there are still some limitations in this study: (1) only target genes of the top three most upregulated and downregulated miRNAs are selected for further enrichment analysis; (2) among the six selected miRNAs, only miR-494-3p and miR-126-3p are selected for experimental verification which may lead to omit some functional miRNAs; (3) a lack of research on detailed molecular mechanisms that miR-494-3p and miR-126-3p regulate invasion and metastasis of HCC is another limitation; (4) related animal studies are deficient in this study.

## Conclusion

In conclusion, we have successfully identified two invasion-metastasis-associated miRNAs (miR-494-3p and miR-126-3p) based on bioinformatic analysis and experimental validation. The present study suggested that miR-494-3p and miR-126-3p play key roles in invasion and metastasis of HCC and can be exploited as two promising targets in treating HCC patients with vascular invasion and metastasis.

## Additional files

**Additional file 1: Figure S1.** The expression levels of miR-1207-5p, miR-1268a, miR-199a-3p and miR-199b-3p in HCC cell lines. (A) The expression of miR-1207-5p in HCC cell lines was compared with that in normal liver cell line (HL7702); (B) the expression of miR-1268a in HCC cell lines was compared with that in HL7702; (C) the expression of miR-199a-3p in HCC cell lines was compared with that in HL7702; (D) the expression of miR-199b-3p in HCC cell lines was compared with that in HL7702. Ns represents no significance; *P < 0.05; **P < 0.01. Error bars represent s.d. for n = 3.

**Additional file 2: Figure S2.** After transfected with mimic at indicated concentration (20 nM, 50 nM and 100 nM), miR-494-3p and miR-126-3p expression levels were significantly increased in both HepG2 and HCCLM3 cell lines. Ns represents no significance; *P < 0.05. Error bars represent s.d. for n = 3. MiR-494-3p and miR-126-3p expression levels were detected using q-PCR at 48 h post-transfection

## Abbreviations

HCC: hepatocellular carcinoma
ECM: extracellular matrix
EMT: epithelial–mesenchymal transition
CSC: cancer stem cell
miRNA: microRNA
mRNA: messenger RNA
GEO: Gene Expression Omnibus
PPI: protein–protein interaction
NCBI: National Center for Biotechnology Information
DAVID: the database for annotation, visualization and integrated discovery
GO: gene ontology
KEGG: Kyoto Encyclopedia of Genes and Genomes
FBS: fetal bovine serum
BP: biological process
CC: cellular component
MF: molecular function
