# Supplementary figures and images for: Identification of invasion-metastasis-associated microRNAs in hepatocellular carcinoma based on bioinformatic analysis and experimental validation
Source: J Transl Med. 2018 Sep 29;16:266. doi: 10.1186/s12967-018-1639-8 (PMC6162949; doi:10.1186/s12967-018-1639-8)

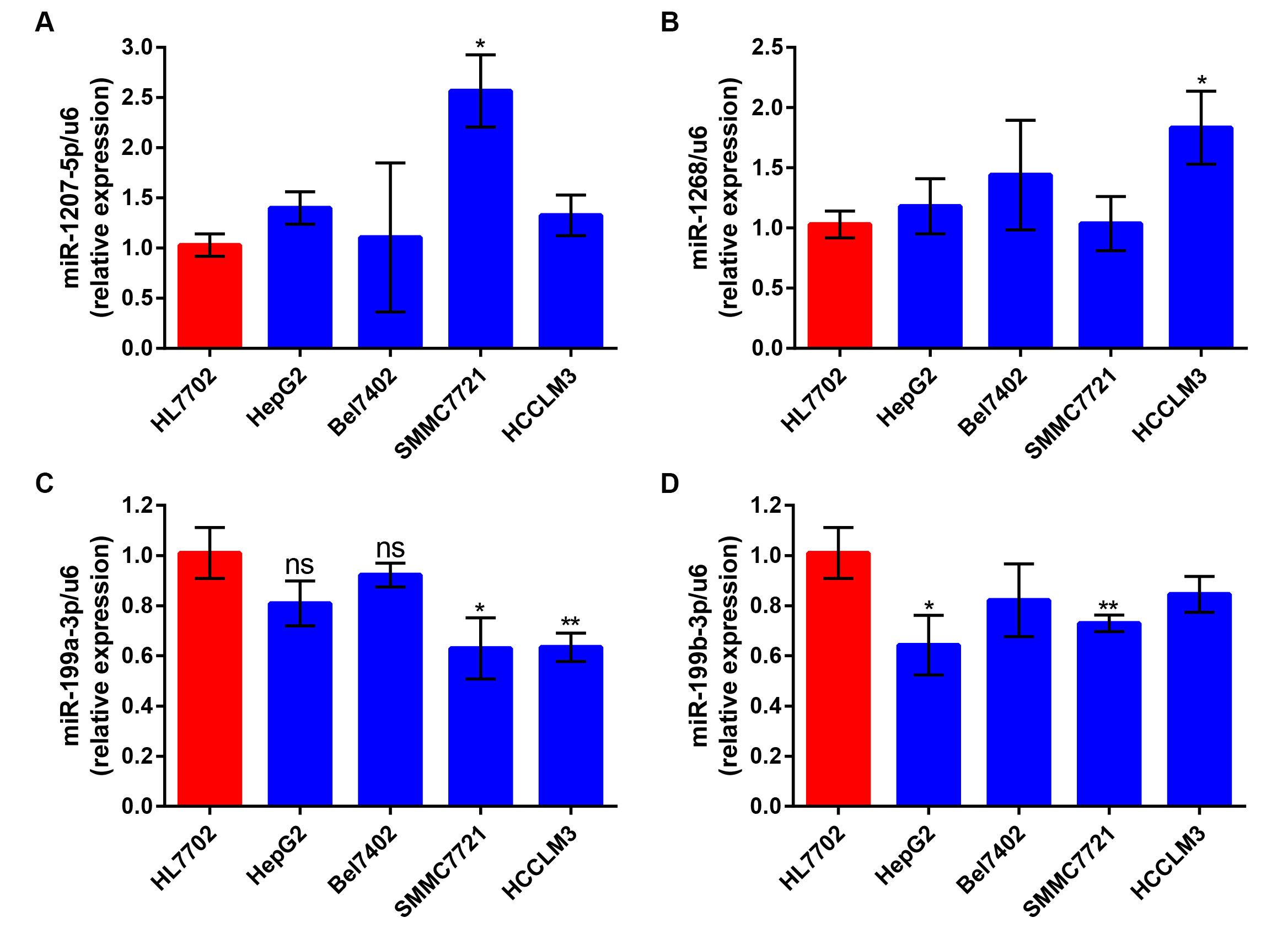

Supplement: Supplementary file 1 — Additional file 1: Figure S1. The expression levels of miR-1207-5p, miR-1268a, miR-199a-3p and miR-199b-3p in HCC cell lines. (A) The expression of miR-1207-5p in HCC cell lines was compared with that in normal liver cell line (HL7702); (B) the expression of miR-1268a in HCC cell lines was compared with that in HL7702; (C) the expression of miR-199a-3p in HCC cell lines was compared with that in HL7702; (D) the expression of miR-199b-3p in HCC cell lines was compared with that in HL7702. Ns represents no significance; *P < 0.05; **P < 0.01. Error bars represent s.d. for n = 3. [file 12967_2018_1639_MOESM1_ESM.jpg]

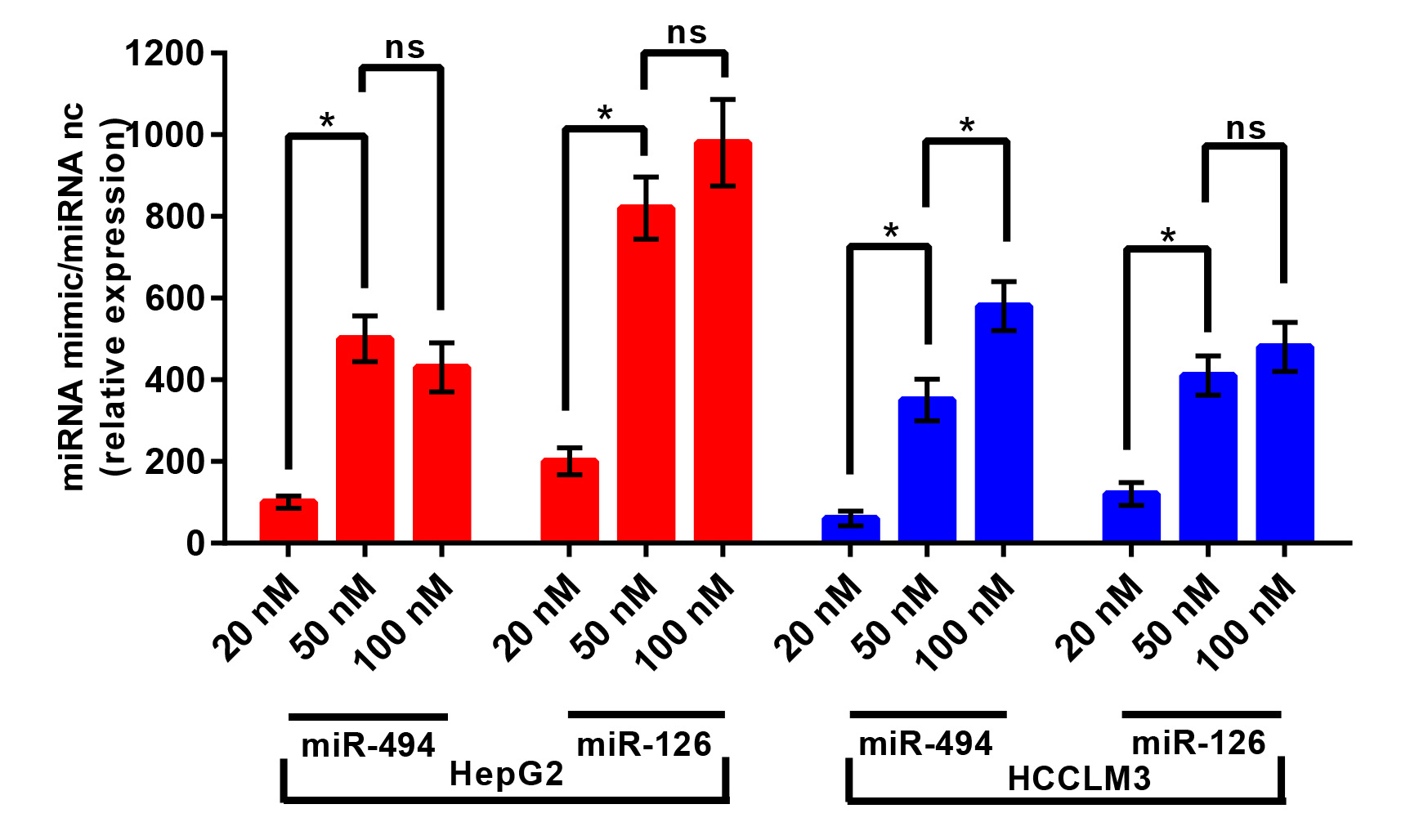

Supplement: Supplementary file 2 — Additional file 2: Figure S2. After transfected with mimic at indicated concentration (20 nM, 50 nM and 100 nM), miR-494-3p and miR-126-3p expression levels were significantly increased in both HepG2 and HCCLM3 cell lines. Ns represents no significance; *P < 0.05. Error bars represent s.d. for n = 3. MiR-494-3p and miR-126-3p expression levels were detected using q-PCR at 48 h post-transfection [file 12967_2018_1639_MOESM2_ESM.jpg]
